# Supplementary material for: Linked symptom monitoring and depression treatment programmes for specialist cancer services: protocol for a mixed-methods implementation study
Source: BMJ Open. 2017 Jul 2;7(6):e016186. doi: 10.1136/bmjopen-2017-016186 (PMC5734371; doi:10.1136/bmjopen-2017-016186)
Supplement: Author's manuscript [file bmjopen-2017-016186.draft_revisions.pdf]

BMJ Open

Introducing linked symptom monitoring and depression treatment programmes in a large cancer service: a mixed-methods implementation study protocol.

Journal:	*BMJ Open*
Manuscript ID	bmjopen-2017-016186
Article Type:	Protocol
Date Submitted by the Author:	30-Jan-2017
Complete List of Authors:	Wanat, Marta; University of Oxford, Psychiatry<br>Walker, J.; University of Oxford, Psychiatry<br>Burke, Katy; University of Oxford, Psychiatry<br>Sevdalis, Nick; King's College London,<br>Richardson, Alison; University of Southampton, Faculty of Health Sciences<br>Mulick, Amy; London School of Hygiene and Tropical Medicine<br>Frost, Chris; London School of Hygiene & Tropical Medicine<br>Sharpe, Michael; University of Oxford, Psychiatry
**Primary Subject Heading**:	Health services research
Secondary Subject Heading:	Health services research
Keywords:	ONCOLOGY, Depression & mood disorders < PSYCHIATRY, implementation process, mixed methods

SCHOLARONE™
Manuscripts

Title: Introducing linked symptom monitoring and depression treatment programmes in a large cancer service: a mixed-methods implementation study protocol.

Authors: Marta Wanat, Jane Walker, Katy Burke, Nick Sevdalis, Alison Richardson, Amy Mulick, Chris Frost, Michael Sharpe.

Author details:

Marta Wanat*, Psychological Medicine Research, University of Oxford Department of Psychiatry, Warneford Hospital, Oxford, UK. marta.wanat@psych.ox.ac.uk, +44 01865 613106

Jane Walker, Psychological Medicine Research, University of Oxford Department of Psychiatry, Warneford Hospital, UK. jane.walker@psych.ox.ac.uk

Katy Burke, Psychological Medicine Research, University of Oxford Department of Psychiatry, Warneford Hospital, Oxford, UK. katharine.burke@psych.ox.ac.uk

Nick Sevdalis, Centre for Implementation Science, King's College London, London, UK. nick.sevdalis@kcl.ac.uk

Alison Richardson, Faculty of Health Sciences, University of Southampton, Southampton, UK. alison.richardson@soton.ac.uk

Amy Mulick, Department of Medical Statistics, London School of Hygiene and Tropical Medicine, London, UK. Amy.Mulick@lshtm.ac.uk

Chris Frost, Department of Medical Statistics, London School of Hygiene and Tropical Medicine, London, UK. Chris.Frost@lshtm.ac.uk

Michael Sharpe, Psychological Medicine Research, University of Oxford Department of Psychiatry, Warneford Hospital, Oxford, UK. michael.sharpe@psych.ox.ac.uk

*corresponding author

Word count: 2778

For peer review only

ABSTRACT

Introduction

There is growing awareness that cancer services need to address patients' wellbeing as well as treating their cancer. During a research programme we developed systematic approaches to (a) monitoring patients' symptoms including depression using a 'Symptom Monitoring Service' and (b) providing treatment for those with major depression using a programme called 'Depression Care for People with Cancer'. Used together, these two programmes were found to be highly effective and cost-effective in clinical trials. The overall aims of this project are to: (1) study the process of introducing the programmes into routine clinical care in a large cancer service, (2) identify the challenges associated with implementation and how these are overcome, (3) determine the programmes' effectiveness in the real world, (4) describe patients' and clinicians' experience of the programmes.

Methods and analysis

This is a mixed methods longitudinal implementation study. We will study the process of implementation in three phases (April 2016-December 2018): "Pre-implementation" (setting up of the new programmes), "Early Implementation" (implementation of the programmes in a small number of clinics) and "Implementation and Maintenance" (implementation in the majority of clinics). We will use the following methods of data collection: (a) contemporaneous logs of the implementation process, (b) interviews with healthcare professionals and managers, (c) interviews with patients, (d) routinely collected clinical data.

Ethics and dissemination

The study has been reviewed by a joint committee of Oxford University Hospitals NHS Foundation Trust Research and Development Department and the University of Oxford's Clinical Trials and Research Governance Department and judged to be service evaluation, not requiring ethics

For peer review only

committee approval. The findings will be crucial for scaling up implementation of the programmes across the UK and will enable us to construct a toolkit for this purpose. We will disseminate our findings in peer-reviewed journals and at relevant national and international conferences.

Keywords Cancer care; symptoms; depression; mixed-methods; RE-AIM; implementation processes

For peer review only

STRENGHTS AND LIMITATIONS

- We will collect data about the process of and barriers and facilitators to implementation from different stakeholders using a variety of methods
- As the implementation of SMS and DCPC in UK cancer centres is explicitly advocated in the 2015 NHS Cancer Taskforce report, the findings of this study will be crucial for scaling up implementation of the programmes across the UK.
- The timeline of the study may be affected by the pace of implementation.
- There may be difficulties in recruiting busy healthcare professionals and managers for interviews.

For peer review only

INTRODUCTION

World-wide, 14.1 million people are diagnosed with cancer each year [1]. Whilst there have been significant advances in anticancer treatments, leading to a longer life expectancy for many patients, there remains a pressing need to also improve patients' quality of life by better management of common cancer-related symptoms such as depression, pain and fatigue [2].

As part of a research programme, we developed two clinical programmes which aimed to improve the quality of life of patients attending cancer services. The first was a systematic approach to the assessment of symptoms, including depression, called the 'Symptom Monitoring Service' (SMS). The second was a system of care for those cancer patients identified as having depression, called 'Depression Care for People with Cancer'(DCPC) [3]. We prioritised the treatment of depression as its management is especially challenging for cancer services. Our research found that, used together, SMS and DCPC are highly effective and cost-effective in improving the lives of those cancer patients who suffer from comorbid depression [4-8].

Symptom Monitoring Service (SMS)

The SMS provides a systematic way of monitoring patients' symptoms, including depression, every time they attend the clinic. Approximately 10% of cancer outpatients have comorbid major depression [9]. Depression substantially reduces patients' quality of life and ability to cope with anticancer treatments, but often goes undetected and untreated [9-11]. Patients using the SMS are assisted to complete brief questionnaires about relevant symptoms using touchscreen computers whilst waiting for their appointment. These questionnaires include symptoms relevant to all clinics (e.g. depressive symptoms and pain) as well as symptoms relevant to specific clinics (e.g. coughing up blood in the lung cancer clinic). The results of these questionnaires are available to the patient's clinician (e.g. oncologist or cancer nurse specialist) before the appointment. The clinician can use this information to: (a) identify patients with severe symptoms; (b) detect changes in patients'

For peer review only

symptoms over time; (c) determine which symptoms require further assessment; (d) ensure that patients receive appropriate treatment for their symptoms. To identify patients with depression, those with a high score on the relevant symptom questionnaire are offered a telephone-delivered diagnostic interview for major depression (the diagnostic term for depression which requires treatment) [12]. Telephone interviews are used because these are more feasible than attempting to achieve interviews in the clinic, are convenient and acceptable to patients, and have been found to have good agreement with face-to-face interviews [13, 14]. Patients identified as having a diagnosis of major depression are offered participation in the linked 'Depression Care for People with Cancer' treatment programme.

Depression Care for People with Cancer (DCPC)

DCPC is a manualised, systematic, multi-component, team-delivered treatment programme for patients with major depression and cancer that is based on the collaborative care model [3, 15]. The treatment team comprises care managers (specially trained cancer nurses) and consultation-liaison psychiatrists who work closely with the patient's GP and oncologist. The care managers establish a therapeutic relationship with the patient, provide education about depression, deliver brief evidence-based psychological treatments (behavioural activation and problem solving therapy) and systematically monitor the patient's progress [16, 17]. The psychiatrists supervise treatment with the aim of achieving and then maintaining specified treatment targets, advise GPs and oncologists on the prescription of antidepressant medication and also provide direct consultations to those patients who are not improving.

The process of implementing a new service

Implementation of these systems has been explicitly advocated in the 2015 NHS Cancer Taskforce report [18]. However, it is well known that the translation from delivering services as part of

For peer review only

research programmes to delivering them in routine clinical practice can be difficult and may lead to innovative services failing to realise their promise [19].

Aim

We therefore plan to study the implementation of SMS and DCPC into the routine clinical care provided by a large cancer service in Oxford, UK, with a view to learning how to facilitate their introduction into other services. We aim to (1) understand the process of implementation, (2) identify the challenges in implementation and how these are overcome, (3) determine the programmes' effectiveness in the real world and (4) describe patients' and clinicians' experience of them.

For peer review only

METHODS AND ANALYSIS

Study design

We will use a mixed-methods design to longitudinally study the process of introducing SMS and DCPC into the Oxford Cancer Centre. The Oxford Cancer Centre is a large cancer service, which is part of Oxford University Hospitals NHS Foundation Trust. It receives approximately 19,000 new referrals per year, has 58 consultant oncologists and comprises inpatient wards, operating theatres, a day hospital and outpatient departments.

The study will focus on three phases: "Pre-implementation" (setting up of the new programmes), "Early Implementation" (implementation in a small number of clinics) and "Implementation and Maintenance" (implementation in the majority of clinics). Our specific aims are described in detail in Table 1. In the pre-implementation phase we will study the process of setting up the new programmes, the challenges encountered during set up and the ways these are addressed. In the early implementation and implementation and maintenance phases, we will describe the challenges encountered and how they are addressed, the real-world effectiveness of the programmes and the experiences of both the patients who have access to SMS and DCPC and of the healthcare professionals involved in them.

For peer review only

Table 1 Study aims related to each phase of implementation

Phase of implementation	Study aim	Relevant Re-aim components	Data collection methods
Pre-implementation	Describe the timeline and process of setting up the new programmes in the cancer service	N/A	Contemporaneous log
	Describe the challenges of setting up the programmes in the cancer service & the ways these are addressed including the experiences of the health professionals and managers involved in set up	N/A	Contemporaneous log, interviews with healthcare professionals & managers
Early implementation	Describe the timeline and process of introducing SMS & DCPC in a small number of clinics	Implementation, Adoption	Contemporaneous log
	Describe the challenges associated with day-to-day running & how these are addressed	Implementation, Adoption, Effectiveness	Contemporaneous log, interviews with healthcare professionals
	Describe the experiences of healthcare professionals involved in SMS & DCPC	Implementation, Adoption, Effectiveness, Reach	Interviews with healthcare professionals
	Describe patients' experience of SMS & DCPC & how these affect their use of them	Effectiveness, Reach	Interviews with patients
	Measure the real-world effectiveness of DCPC by comparing patient outcomes with those achieved in clinical trials	Effectiveness	Routine clinical data
	Measure the proportion & characteristics of the target population who have access to & who use SMS & DCPC	Reach	Routine clinical data
Implementation and maintenance	Describe the timeline and process of the introduction of SMS & DCPC in the majority of clinics	Adoption, Maintenance	Contemporaneous log
	Describe the challenges associated with implementation and long-term maintenance & how these are addressed	Implementation, Maintenance	Contemporaneous log, interviews with healthcare professionals
	Describe the challenges of delivering SMS & DCPC as intended & adaptations made to the programmes	Implementation, maintenance	Contemporaneous log, interviews with healthcare professionals
	Describe the experiences of healthcare professionals involved in SMS & DCPC	Adoption, Effectiveness	Interviews with healthcare professionals
	Describe patients' experience of SMS & DCPC & how these affect their use of them	Effectiveness, Reach	Interviews with patients
	Measure the real-world effectiveness of DCPC over time by comparing patient outcomes with those achieved in clinical trials	Effectiveness, Maintenance	Routine clinical data
	Determine the extent to which SMS & DCPC are delivered as intended	Implementation	Routine clinical data
	Measure the proportion & characteristics of the target population who have access to & who use SMS & DCPC over time	Reach	Routine clinical data
	Measure the cost of delivering SMS & DCPC	Implementation	Routine clinical data

For peer review only

We will use the following methods of data collection: (a) contemporaneous logs of the implementation process, (b) interviews with healthcare professionals and managers, (c) interviews with patients, (d) routine clinical data. Data collection in the Early Implementation and Implementation and Maintenance phases will be guided by the RE-AIM (Reach, Effectiveness, Adoption, Implementation, Maintenance) framework [20, 21].

Pre-implementation phase

Contemporaneous log

A contemporaneous events log will be used by the study team to record any activities and events related to setting up the new programmes in the cancer service and will include regular correspondence from staff involved in setting up the service [22]. Sample activities and events to be included are recruitment and training events, and meetings with clinical managers and IT experts. Each entry to the log will include information on the date of the action or event, the people involved and its effects on the delivery of the programmes.

Interviews with healthcare professionals and managers

We will recruit and interview 10-15 healthcare professionals and managers who are involved in setting up SMS and DCPC in the cancer service. Based on similar studies, we anticipate this sample size will be sufficient to reach data saturation [23]. Interviews will take approximately 30 minutes, will be audio-recorded and transcribed verbatim and will be conducted at the participants' preferred location or by telephone. An interview topic guide will focus on participants' views of the process of setting up the programmes.

For peer review only

Analysis

A timeline of events that occur during set up will be drawn using the contemporaneous log. Data from the log will then be analysed using content analysis, which will involve data familiarisation and coding. Codes will be developed focusing on the process of setting up the programmes, the associated challenges and the ways that these are addressed. The codes developed from the contemporaneous log will be applied to the transcripts of interviews with healthcare professionals and managers and will be further refined. These codes will be then sorted into subcategories and categories.

Early Implementation and Implementation and Maintenance phases

Contemporaneous log

We will record and use data in a contemporaneous events log in the same away as in the pre-implementation phase.

Interviews with healthcare professionals and managers

We will recruit 20-30 healthcare professionals and managers during the Early Implementation and Implementation and Maintenance phases. These will include psychiatrists and care managers involved in the delivery of the SMS and DCPC, and oncologists, cancer nurse specialists and GPs who make up the wider collaborative care team. Based on similar studies, we anticipate this sample size will be sufficient to reach data saturation [23]. Interviews will take approximately 30 minutes, will be audio-recorded and transcribed verbatim and will be conducted at the participant's preferred location or by telephone. The interview topic guide will focus on the participants' experience of the new programmes.

For peer review only

Interviews with patients

We will recruit and interview 40-60 patients during the Early Implementation and Implementation and Maintenance phases. These will include patients who do not use the first stage of the SMS service (n=10-15), who use the SMS service (n=10-15), who do not use the DCPC treatment programme (n=10-15), and who use the DCPC treatment programme (n=10-15). We will exclude patients who are unable to provide informed consent to participate or do not understand and speak English sufficiently to participate in a qualitative interview. Interviews will take between 30-60 minutes, will be audio-recorded and transcribed verbatim and will be conducted at the participant's preferred location or by telephone. The interview topic guide will focus on patients' understanding and views of the SMS and DCPC.

Routine clinical data

We will obtain the following data from the cancer centre: the number and characteristics of clinics with and without the new programmes over time, the proportion of patients who do and do not receive each part of the programmes and their characteristics, the costs of delivering SMS and DCPC, adherence to the SMS and DCPC manuals, outcomes of patients who receive the programmes and description of any relevant adverse clinical events.

Analysis

Data recorded in the contemporaneous log related to the introduction of SMS and DCPC in clinics will be used to draw a timeline across the Early Implementation and Implementation and Maintenance phases. Then, data from the contemporaneous log will be analysed using content analysis, which will involve data familiarisation and coding. Codes will be developed focusing on: the process of implementation, challenges associated with day-to-day running of SMS and DCPC, challenges associated with implementation and long-term maintenance, challenges associated with delivering SMS and DCPC as intended, and the ways that these challenges are addressed. Codes

For peer review only

developed from the contemporaneous log will be applied to selected relevant excerpts from the transcripts of qualitative interviews with healthcare professionals. These codes will be further refined and then sorted into subcategories and categories.

Data generated from the qualitative interviews with patients and with healthcare professionals involved in SMS and DCPC will be analysed using thematic analysis to describe their experiences [24]. The stages of the analysis will include data familiarisation, coding and development of themes. Data collection and analysis will take place simultaneously to allow the exploration of themes from the initial interviews in subsequent ones. In order to minimise subjective biases, the coding will be discussed among members of the study team.

We will analyse the routine clinical data using descriptive statistics in order to summarise: the real-world effectiveness of DCPC over time (including benchmarking against the patient outcomes in our clinical trials), the proportion and characteristics of the target population who have access to and who use SMS and DCPC over time, the fidelity of the delivery of SMS and DCPC, adherence of patients with each component of the DCPC and the cost of the new services. We will use data from qualitative interviews to understand these results, for example how challenges in the adherence to the protocol may influence the effectiveness of the service.

Ethics and dissemination

The study has been reviewed by a joint committee of Oxford University Hospitals NHS Foundation Trust Research and Development Department and the University of Oxford's Clinical Trials and Research Governance Department and been judged to be service evaluation, not requiring ethics committee approval. The study has been registered with the Oxford University Hospitals NHS Foundation Trust service evaluation and clinical audit team. The results of the study will be

For peer review only

disseminated via presentations at local, national and international conferences, peer-reviewed journals and workshops with stakeholders. The implementation of SMS and DCPC in UK cancer centres is explicitly advocated in the 2015 NHS Cancer Taskforce report [10]. The findings from the study will be crucial for scaling up implementation and will form part of a toolkit for doing this.

For peer review only

DISCUSSION

Our overall aim is to study the process of setting up and introducing two new linked clinical programmes (SMS and DCPC) in a large UK cancer service, to describe the challenges associated with their implementation and how these are addressed, to measure their effectiveness in the real world and to describe patients' and clinicians' experience of using them. Whilst these programmes have already been successfully implemented in the context of a large research programme and have been found to be effective and cost-effective in clinical trials, this is first time that they have been implemented in a non-research setting.

The Early Implementation and Implementation and Maintenance phases of our study will use the RE-AIM framework to guide data collection, analysis and reporting of findings. The authors of this framework have recently highlighted the importance of collecting data in relation to all components of RE-AIM framework and especially qualitative data about patients not using the service [21]. Our study follows both of these recommendations.

We anticipate a number of challenges in conducting this research. Firstly, the timeline of the study will be affected by the pace of implementation. We have divided the study into three phases to minimise delays to data collection. Secondly, there may be difficulties in recruiting busy healthcare professionals and managers for interviews. We have mitigated this risk by working closely with healthcare professionals and managers to identify research questions which are of interest to them. Thirdly, this study involves multiple methods of data collection. We will make sure that we integrate qualitative and quantitative findings to further our understanding of the challenges associated with the implementation of the clinical programmes. Finally, we will also make sure that the research team works sufficiently close with the implementation team to be able to gather data whilst remaining independent enough to portray the problems accurately [25].

For peer review only

This study will provide us with robust new knowledge, essential for service planning, about the process of implementing these two linked programmes and the challenges to implementation success. We will also find out what patients and clinicians think of SMS and DCPC and how they can be further improved. The findings of this study will be crucial for scaling up implementation of the programmes across the UK and will enable us to construct a toolkit for this purpose. More widely, the study findings will further our understanding of the mechanisms involved in implementing new systems of care within an established clinical service.

For peer review only

REFERENCES

1. Torre, L.A., et al., *Global cancer statistics, 2012.* CA Cancer J Clin, 2015. **65**(2): p. 87-108.
2. Richardson, A., et al., *Knowledge, ignorance and priorities for research in key areas of cancer survivorship: findings from a scoping review.* Br J Cancer, 2011. **105 Suppl 1**: p. S82-94.
3. Walker, J. and M. Sharpe, *Depression care for people with cancer: a collaborative care intervention.* General hospital psychiatry, 2009. **31**(5): p. 436-441.
4. Strong, V., et al., *Management of depression for people with cancer (SMaRT oncology 1): a randomised trial.* The Lancet, 2008. **372**(9632): p. 40-48.
5. Sharpe, M., et al., *Integrated collaborative care for comorbid major depression in patients with cancer (SMaRT Oncology-2): a multicentre randomised controlled effectiveness trial.* The Lancet, 2014. **384**(9948): p. 1099-1108.
6. Walker, J., et al., *Integrated collaborative care for major depression comorbid with a poor prognosis cancer (SMaRT Oncology-3): a multicentre randomised controlled trial in patients with lung cancer.* The Lancet Oncology, 2014. **15**(10): p. 1168-1176.
7. Duarte, A., et al., *Cost-effectiveness of integrated collaborative care for comorbid major depression in patients with cancer.* J Psychosom Res, 2015. **79**(6): p. 465-70.
8. Walker, S., et al., *Cost-effectiveness of combining systematic identification and treatment of co-morbid major depression for people with chronic diseases: the example of cancer.* Psychol Med, 2014. **44**(7): p. 1451-60.
9. Walker, J., et al., *Prevalence, associations, and adequacy of treatment of major depression in patients with cancer: a cross-sectional analysis of routinely collected clinical data.* Lancet Psychiatry, 2014. **1**(5): p. 343-50.
10. Kroenke, K., et al., *The association of depression and pain with health-related quality of life, disability, and health care use in cancer patients.* J Pain Symptom Manage, 2010. **40**(3): p. 327-341.

For peer review only

11. Colleoni, M., et al., *Depression and degree of acceptance of adjuvant cytotoxic drugs.* Lancet, 2000. **356**(9238): p. 1326-1327.

12. American Psychiatric Association, *Diagnostic and Statistical Manual of Mental Mental Disorders fifth edition, DSM-5*. 2013: American Psychiatric Association.

13. Simon, G.E., D. Revicki, and M. VonKorff, *Telephone assessment of depression severity.* Journal of psychiatric research, 1993. **27**(3): p. 247-252.

14. Allen, K., A. Cull, and M. Sharpe, *Diagnosing major depression in medical outpatients: acceptability of telephone interviews.* J.Psychosom.Res., 2003. **55**(4): p. 385-387.

15. Katon, W., *Collaborative depression care models: from development to dissemination.* Am J Prev Med, 2012. **42**(5): p. 550-2.

16. Hopko, D.R., et al., *Contemporary behavioral activation treatments for depression: Procedures, principles, and progress.* Clinical psychology review, 2003. **23**(5): p. 699-717.

17. Mynors-Wallis, L., *Problem-solving treatment for anxiety and depression: A practical guide*. 2005: Oxford University Press, USA.

18. Independent Cancer Taskforce, *Achieving world-class cancer outcomes. A strategy for England 2015-2020*. 2015: London.

19. Grol, R. and J. Grimshaw, *From best evidence to best practice: effective implementation of change in patients' care.* The lancet, 2003. **362**(9391): p. 1225-1230.

20. Glasgow, R.E., T.M. Vogt, and S.M. Boles, *Evaluating the public health impact of health promotion interventions: the RE-AIM framework.* American journal of public health, 1999. **89**(9): p. 1322-1327.

21. Kessler, R.S., et al., *What Does It Mean to ''Employ''the RE-AIM Model?* Evaluation & the health professions, 2012: p. 0163278712446066.

22. Pannick, S., N. Sevdalis, and T. Athanasiou, *Beyond clinical engagement: a pragmatic model for quality improvement interventions, aligning clinical and managerial priorities.* BMJ Quality & Safety, 2015.

For peer review only

23. Francis, J.J., et al., *What is an adequate sample size? Operationalising data saturation for theory-based interview studies.* Psychology and Health, 2010. **25**(10): p. 1229-1245.

24. Braun, V. and V. Clarke, *Using thematic analysis in psychology.* Qualitative research in psychology, 2006. **3**(2): p. 77-101.

25. Moore, G.F., et al., *Process evaluation of complex interventions: Medical Research Council guidance.* bmj, 2015. **350**: p. h1258.

For peer review only

AUTHOR'S CONTRIBUTIONS

Marta Wanat: study design and conduct; manuscript writing.

Jane Walker: study conception, obtained funding, study design and conduct, manuscript writing.

Katy Burke: advised on the study design, manuscript review.

Nick Sevdalis: advised on the study design, manuscript review

Alison Richardson: advised on the study design, manuscript review.

Amy Mulick: advised on the study design, manuscript review.

Chris Frost: advised on the study design, manuscript review.

Michael Sharpe: study conception, obtained funding, study design and conduct, manuscript writing.

FUNDING

The study is funded by the National Institute for Health Research (NIHR) Collaboration for Leadership in Applied Health Research and Care Oxford at Oxford Health NHS Foundation Trust. Sevdalis' research is supported by the National Institute for Health Research (NIHR) Collaboration for Leadership in Applied Health Research and Care South London at King's College Hospital NHS Foundation Trust. Sevdalis is a member of King's Improvement Science, which is part of the NIHR CLAHRC South London and comprises a specialist team of improvement scientists and senior researchers based at King's College London. Its work is funded by King's Health Partners (Guy's and St Thomas' NHS Foundation Trust, King's College Hospital NHS Foundation Trust, King's College London and South London and Maudsley NHS Foundation Trust), Guy's and St Thomas' Charity, the Maudsley Charity and the Health Foundation. The views expressed are those of the authors and not necessarily those of the NHS, the NIHR, or the Department of Health.

COMPETING INTEREST

Sevdalis is the director of London Training & Safety Solutions Ltd, which consults on team training, human factors and patient safety.

For peer review only

For peer review only

BMJ Open

Linked symptom monitoring and depression treatment programmes for specialist cancer services: Protocol for a mixed-methods implementation study.

Journal:	*BMJ Open*
Manuscript ID	bmjopen-2017-016186.R1
Article Type:	Protocol
Date Submitted by the Author:	15-May-2017
Complete List of Authors:	Wanat, Marta; University of Oxford, Psychiatry<br>Walker, J.; University of Oxford, Psychiatry<br>Burke, Katy; University of Oxford, Psychiatry<br>Sevdalis, Nick; King's College London,<br>Richardson, Alison; University of Southampton, Faculty of Health Sciences<br>Mulick, Amy; London School of Hygiene and Tropical Medicine<br>Frost, Chris; London School of Hygiene & Tropical Medicine<br>Sharpe, Michael; University of Oxford, Psychiatry
**Primary Subject Heading**:	Health services research
Secondary Subject Heading:	Health services research
Keywords:	ONCOLOGY, Depression & mood disorders < PSYCHIATRY, implementation process, mixed methods

SCHOLARONE™
Manuscripts

For peer review only

Title: Linked symptom monitoring and depression treatment programmes for specialist cancer services: Protocol for a mixed-methods implementation study.

Authors: Marta Wanat, Jane Walker, Katy Burke, Nick Sevdalis, Alison Richardson, Amy Mulick, Chris Frost, Michael Sharpe.

Author details:

Marta Wanat*, Psychological Medicine Research, University of Oxford Department of Psychiatry, Warneford Hospital, Oxford, UK. marta.wanat@psych.ox.ac.uk, +44 01865 613106

Jane Walker, Psychological Medicine Research, University of Oxford Department of Psychiatry, Warneford Hospital, UK. jane.walker@psych.ox.ac.uk

Katy Burke, Psychological Medicine Research, University of Oxford Department of Psychiatry, Warneford Hospital, Oxford, UK. katharine.burke@psych.ox.ac.uk

Nick Sevdalis, Centre for Implementation Science, King's College London, London, UK. nick.sevdalis@kcl.ac.uk

Alison Richardson, Faculty of Health Sciences, University of Southampton, Southampton, UK. alison.richardson@soton.ac.uk

Amy Mulick, Department of Medical Statistics, London School of Hygiene and Tropical Medicine, London, UK. Amy.Mulick@lshtm.ac.uk

Chris Frost, Department of Medical Statistics, London School of Hygiene and Tropical Medicine, London, UK. Chris.Frost@lshtm.ac.uk

Michael Sharpe, Psychological Medicine Research, University of Oxford Department of Psychiatry, Warneford Hospital, Oxford, UK. michael.sharpe@psych.ox.ac.uk

*corresponding author

Word count: 2778

For peer review only

For peer review only - http://bmjopen.bmj.com/site/about/guidelines.xhtml

ABSTRACT

Introduction

There is growing awareness that cancer services need to address patients' wellbeing as well as treating their cancer. We developed systematic approaches to (a) monitoring patients' symptoms including depression using a 'Symptom Monitoring Service' and (b) providing treatment for those with major depression using a programme called 'Depression Care for People with Cancer'. Used together, these two programmes were found to be highly effective and cost-effective in clinical trials. The overall aims of this project are to: (1) study the process of introducing these programmes into routine clinical care in a large cancer service, (2) identify the challenges associated with implementation and how these are overcome, (3) determine their effectiveness in a routine non-research setting, (4) describe patients' and clinicians' experience of the programmes.

Methods and analysis

This is a mixed methods longitudinal implementation study. We will study the process of implementation in three phases (April 2016-December 2018): "Pre-implementation" (setting up of the new programmes), "Early Implementation" (implementation of the programmes in a small number of clinics) and "Implementation and Maintenance" (implementation in the majority of clinics). We will use the following methods of data collection: (a) contemporaneous logs of the implementation process, (b) interviews with healthcare professionals and managers, (c) interviews with patients, (d) routinely collected clinical data.

Ethics and dissemination

The study has been reviewed by a joint committee of Oxford University Hospitals NHS Foundation Trust Research and Development Department and the University of Oxford's Clinical Trials and Research Governance Department and judged to be service evaluation, not requiring ethics committee approval. The findings of this study will guide the scaling up implementation of the

For peer review only - http://bmjopen.bmj.com/site/about/guidelines.xhtml

programmes across the UK and will enable us to construct an implementation toolkit. We will disseminate our findings in publications and at relevant national and international conferences.

Keywords Cancer care; symptoms; depression; mixed-methods; RE-AIM; implementation processes

For peer review only

For peer review only - http://bmjopen.bmj.com/site/about/guidelines.xhtml

STRENGTHS AND LIMITATIONS

- The mixed-methods design of this study will produce a comprehensive summary of the process of, and barriers and facilitators to, implementation.
- The implementation of our depression screening and treatment programmes was explicitly advocated in the 2015 NHS Cancer Taskforce report, and the findings of this study will inform the process of implementation in other centres.
- The timeline of the study may be affected by the pace of implementation.
- The study findings may not be readily generalizable to other healthcare systems.

For peer review only

INTRODUCTION

World-wide, 14.1 million people are diagnosed with cancer each year [1]. Whilst there have been significant advances in anticancer treatments, leading to a longer life expectancy for many patients, there remains a pressing need to also improve patients' quality of life by better management of common cancer-related symptoms such as depression, pain and fatigue [2].

As part of a research programme, we developed two clinical programmes which aimed to improve the quality of life of patients attending cancer services. The first was a systematic approach to the assessment of symptoms, including depression, called the 'Symptom Monitoring Service' (SMS). The second was a system of care for those cancer patients identified as having depression, called 'Depression Care for People with Cancer'(DCPC) [3]. We prioritised the treatment of depression as its management is especially challenging for cancer services. Our research found that, used together, SMS and DCPC are highly effective and cost-effective in improving the lives of those cancer patients who suffer from comorbid depression [4-8].

Symptom Monitoring Service (SMS)

The SMS provides a systematic way of monitoring patients' symptoms, including depression, every time they attend the clinic. Approximately 10% of cancer outpatients have comorbid major depression [9]. Depression substantially reduces patients' quality of life and ability to cope with anticancer treatments, but often goes undetected and untreated [9-11]. Patients using the SMS are assisted to complete brief questionnaires about relevant symptoms using touchscreen computers whilst waiting for their appointment. These questionnaires include symptoms relevant to all clinics (e.g. depressive symptoms and pain) as well as symptoms relevant to specific clinics (e.g. coughing up blood in the lung cancer clinic). The results of these questionnaires are available to the patient's clinician (e.g. oncologist or cancer nurse specialist) before the appointment. The clinician can use this information to: (a) identify patients with severe symptoms; (b) detect changes in patients'

For peer review only

symptoms over time; (c) determine which symptoms require further assessment; (d) ensure that patients receive appropriate treatment for their symptoms. To identify patients with major depression (the diagnostic term for depression which requires treatment), those with a high score on the relevant symptom questionnaire are offered a telephone-delivered diagnostic interview [12]. These semi-structured clinical interviews are based on the major depression section of the Structured Clinical Interview for the Diagnostic and Statistical Manual of Mental Disorders) [13]. Telephone interviews are used because these are more feasible than attempting to achieve interviews in the clinic, are convenient and acceptable to patients, and have been found to have good agreement with face-to-face interviews [14, 15]. The SMS is delivered by a team of symptom monitoring assistants, trained and supervised by consultation-liaison psychiatrists; further details are given in our previous publication[16]. Patients identified as having a diagnosis of major depression are offered participation in the linked 'Depression Care for People with Cancer' treatment programme.

Depression Care for People with Cancer (DCPC)

DCPC is a manualised, systematic, multi-component, team-delivered treatment programme for patients with cancer and major depression that is based on the collaborative care model [3, 17]. The treatment team comprises care managers (specially trained cancer nurses) and consultation-liaison psychiatrists who work closely with the patient's GP and oncologist. The care managers establish a therapeutic relationship with the patient, provide education about depression, deliver brief evidence-based psychological treatments (behavioural activation and problem solving therapy) and systematically monitor the patient's progress [18, 19]. The psychiatrists supervise treatment with the aim of achieving and then maintaining specified treatment targets, advise GPs and oncologists on the prescription of antidepressant medication and also provide direct consultations to those patients who are not improving.

For peer review only - http://bmjopen.bmj.com/site/about/guidelines.xhtml

The process of implementing a new service

Implementation of these systems has been explicitly advocated in the 2015 NHS Cancer Taskforce report [20]. However, it is well known that the translation from delivering services as part of research programmes to delivering them in routine clinical practice can be difficult and may lead to innovative services failing to realise their promise [21].

Aim

Our aim is to study the implementation of SMS and DCPC into the routine clinical care provided by a large cancer service in Oxford, UK, with a view to learning how best to facilitate the introduction of these programmes into other cancer services. We will: (1) describe the process of implementation, (2) identify the challenges in implementation and how these are overcome, (3) determine the programmes' effectiveness in clinical practice and (4) describe patients' and clinicians' experience of them. Detailed aims are described in Table 1.

For peer review only

For peer review only - http://bmjopen.bmj.com/site/about/guidelines.xhtml

METHODS AND ANALYSIS

Study design

We will use a mixed-methods design to longitudinally study the process of introducing SMS and DCPC into the Oxford Cancer Centre. The Oxford Cancer Centre is a large cancer service, which is part of Oxford University Hospitals NHS Foundation Trust. It receives approximately 19,000 new referrals per year, has 58 consultant oncologists and comprises inpatient wards, operating theatres, a day hospital and outpatient departments.

The study will focus on three phases: "Pre-implementation" (setting up of the new programmes), "Early Implementation" (implementation in a small number of clinics) and "Implementation and Maintenance" (implementation in the majority of clinics). Our specific aims are described in detail in Table 1. In the pre-implementation phase we will study the process of setting up the new programmes, the challenges encountered during set up and the ways these are addressed. In the early implementation and implementation and maintenance phases, we will describe the challenges encountered and how they are addressed, the effectiveness of the programmes in clinical practice and the experiences of both the patients who have access to SMS and DCPC and of the healthcare professionals involved in them.

For peer review only

For peer review only - http://bmjopen.bmj.com/site/about/guidelines.xhtml

Table 1 Study aims related to each phase of implementation

Phase of implementation	Study aim	Relevant Re-aim components	Data collection methods
Pre-implementation	Describe the timeline and process of setting up the new programmes in the cancer service	N/A	Contemporaneous log
	Describe the challenges of setting up the programmes in the cancer service & the ways these are addressed including the experiences of the health professionals and managers involved in set up	N/A	Contemporaneous log, interviews with healthcare professionals & managers
Early implementation	Describe the timeline and process of introducing SMS & DCPC in a small number of clinics	Implementation, Adoption	Contemporaneous log
	Describe the challenges associated with day-to-day running & how these are addressed	Implementation, Adoption, Effectiveness	Contemporaneous log, interviews with healthcare professionals
	Describe the experiences of healthcare professionals involved in SMS & DCPC	Implementation, Adoption, Effectiveness, Reach	Interviews with healthcare professionals
	Describe patients' experience of SMS & DCPC & how these affect their use of them	Effectiveness, Reach	Interviews with patients
	Measure the clinical effectiveness of DCPC by comparing patient outcomes with those achieved in clinical trials	Effectiveness	Routine clinical data
	Measure the proportion & characteristics of the target population who have access to & who use SMS & DCPC	Reach	Routine clinical data
Implementation and maintenance	Describe the timeline and process of the introduction of SMS & DCPC in the majority of clinics	Adoption, Maintenance	Contemporaneous log
	Describe the challenges associated with implementation and long-term maintenance & how these are addressed	Implementation, Maintenance	Contemporaneous log, interviews with healthcare professionals
	Describe the challenges of delivering SMS & DCPC as intended & adaptations made to the programmes	Implementation, maintenance	Contemporaneous log, interviews with healthcare professionals
	Describe the experiences of healthcare professionals involved in SMS & DCPC	Adoption, Effectiveness	Interviews with healthcare professionals
	Describe patients' experience of SMS & DCPC & how these affect their use of them	Effectiveness, Reach	Interviews with patients
	Measure the effectiveness of DCPC over time by comparing patient outcomes with those achieved in clinical trials	Effectiveness, Maintenance	Routine clinical data
	Determine the extent to which SMS & DCPC are delivered as intended	Implementation	Routine clinical data
	Measure the proportion & characteristics of the target population who have access to & who use SMS & DCPC over time	Reach	Routine clinical data
	Measure the cost of delivering SMS & DCPC	Implementation	Routine clinical data

For peer review only

We will use the following methods of data collection: (a) contemporaneous logs of the implementation process, (b) interviews with healthcare professionals and managers, (c) interviews with patients, (d) routine clinical data. Data collection in the Early Implementation and Implementation and Maintenance phases will be guided by the RE-AIM (Reach, Effectiveness, Adoption, Implementation, Maintenance) framework [22, 23].

Pre-implementation phase

Contemporaneous log

A contemporaneous events log will be used by the study team to record any activities and events related to setting up the new programmes in the cancer service and will include regular correspondence from staff involved in setting up the service [24]. Sample activities and events to be included are recruitment and training events, and meetings with clinical managers and IT experts. Each entry to the log will include information on the date of the action or event, the people involved and its effects on the delivery of the programmes.

Interviews with healthcare professionals and managers

We will recruit and interview 10-15 healthcare professionals and managers who are involved in setting up SMS and DCPC in the cancer service. Based on similar studies, we anticipate this sample size will be sufficient to reach data saturation [25]. Interviews will take approximately 30 minutes, will be audio-recorded and transcribed verbatim and will be conducted at the participants' preferred location or by telephone. Interviews will be semi-structured following a topic guide which will focus on participants' views of the process of setting up the programmes. They will conducted by a trained member of the study team with previous experience of interviewing healthcare professionals.

For peer review only - http://bmjopen.bmj.com/site/about/guidelines.xhtml

Analysis

A timeline of events that occur during set up will be drawn using the contemporaneous log. Data from the log will then be analysed using content analysis, which will involve data familiarisation and coding. Codes will be developed focusing on the process of setting up the programmes, the associated challenges and the ways that these are addressed. The codes developed from the contemporaneous log will be applied to the transcripts of interviews with healthcare professionals and managers and will be further refined. These codes will be then sorted into subcategories and categories.

Early Implementation and Implementation and Maintenance phases

Contemporaneous log

We will record and use data in a contemporaneous events log in the same away as in the pre-implementation phase. This will allow us to describe the time taken for each step of implementation.

Interviews with healthcare professionals and managers

We will recruit 20-30 healthcare professionals and managers during the Early Implementation and Implementation and Maintenance phases. These will include psychiatrists and care managers involved in the delivery of the SMS and DCPC, and oncologists, cancer nurse specialists and GPs who make up the wider collaborative care team. Based on similar studies, we anticipate this sample size will be sufficient to reach data saturation [25]. Semi-structured Interviews will take approximately 30 minutes, will be audio-recorded and transcribed verbatim and will be conducted at the participant's preferred location or by telephone. Interviews will be conducted by a trained member of the study team with previous experience of interviewing healthcare professionals. The interview topic guide will focus on the participants' experience of the new programmes.

For peer review only - http://bmjopen.bmj.com/site/about/guidelines.xhtml

For peer review only

Interviews with patients

We will recruit and interview 40-60 patients during the Early Implementation and Implementation and Maintenance phases. These will include patients who do not use the first stage of the SMS service (n=10-15), who use the SMS service (n=10-15), who do not use the DCPC treatment programme (n=10-15), and who use the DCPC treatment programme (n=10-15). We will exclude patients who are unable to provide informed consent to participate or do not understand and speak English sufficiently to participate in a qualitative interview. Interviews will take between 30-60 minutes, will be audio-recorded and transcribed verbatim and will be conducted at the participant's preferred location or by telephone. Interviews will be conducted by a trained member of the study team with previous experience of interviewing patients with cancer. The interview topic guide will focus on patients' understanding and views of the SMS and DCPC.

For peer review only

Routine clinical data

We will obtain the following data from the cancer centre: the number and characteristics of clinics with and without the new programmes over time, the proportion of patients who do and do not receive each part of the programmes and their characteristics, the staff and other resources required and costs of delivering SMS and DCPC, adherence to the SMS and DCPC manuals including diagnostic accuracy, outcomes of patients who receive the programmes and description of any relevant adverse clinical events.

Analysis

Data recorded in the contemporaneous log related to the introduction of SMS and DCPC in clinics will be used to draw a timeline across the Early Implementation and Implementation and Maintenance phases. Then, data from the contemporaneous log will be analysed using content analysis, which will involve data familiarisation and coding. Codes will be developed focusing on: the process of implementation, challenges associated with day-to-day running of SMS and DCPC,

For peer review only - http://bmjopen.bmj.com/site/about/guidelines.xhtml

challenges associated with implementation and long-term maintenance, challenges associated with delivering SMS and DCPC as intended, and the ways that these challenges are addressed. Codes developed from the contemporaneous log will be applied to selected relevant excerpts from the transcripts of qualitative interviews with healthcare professionals. These codes will be further refined and then sorted into subcategories and categories.

Data generated from the qualitative interviews with patients and with healthcare professionals involved in SMS and DCPC will be analysed using thematic analysis to describe their experiences [26]. The stages of the analysis will include data familiarisation, coding and development of themes. Data collection and analysis will take place simultaneously to allow the exploration of themes from the initial interviews in subsequent ones. In order to minimise subjective biases, the coding will be discussed among members of the study team.

We will analyse the routine clinical data using descriptive statistics in order to summarise: the effectiveness of DCPC over time (including benchmarking against the patient outcomes in our clinical trials), the proportion and characteristics of the target population who have access to and who use SMS and DCPC over time, the fidelity of the delivery of SMS and DCPC, adherence of patients with each component of the DCPC and the cost of the new services. We will use data from qualitative interviews to understand these results, for example how challenges in the adherence to the protocol may influence the effectiveness of the service.

Ethics and dissemination

The study has been reviewed by a joint committee of Oxford University Hospitals NHS Foundation Trust Research and Development Department and the University of Oxford's Clinical Trials and Research Governance Department and been judged to be service evaluation, not requiring ethics

For peer review only - http://bmjopen.bmj.com/site/about/guidelines.xhtml

committee approval. The study has been registered with the Oxford University Hospitals NHS Foundation Trust service evaluation and clinical audit team. The results of the study will be disseminated via presentations at local, national and international conferences, peer-reviewed journals and workshops with stakeholders. The implementation of SMS and DCPC in UK cancer centres is explicitly advocated in the 2015 NHS Cancer Taskforce report [10]. The findings from the study will be crucial for scaling up implementation and will form part of a toolkit for doing this.

For peer review only

For peer review only - http://bmjopen.bmj.com/site/about/guidelines.xhtml

DISCUSSION

Our overall aim is to study the process of setting up and introducing two new linked clinical programmes (SMS and DCPC) in a large UK cancer service, to describe the challenges associated with their implementation and how these are addressed, to measure their effectiveness in clinical practice and to describe patients' and clinicians' experience of using them. Whilst these programmes have already been successfully implemented in the context of a large research programme and have been found to be effective and cost-effective in clinical trials, this is first time that they have been implemented in a non-research setting.

The Early Implementation and Implementation and Maintenance phases of our study will use the RE-AIM framework to guide data collection, analysis and reporting of findings. The authors of this framework have recently highlighted the importance of collecting data in relation to all components of RE-AIM framework and especially qualitative data about patients not using the service [23]. Our study follows both of these recommendations.

We anticipate a number of challenges in conducting this research. Firstly, the timeline of the study will be affected by the pace of implementation. We have divided the study into three phases to minimise delays to data collection. Secondly, there may be difficulties in recruiting busy healthcare professionals and managers for interviews. We have mitigated this risk by working closely with healthcare professionals and managers to identify research questions which are of interest to them. Thirdly, this study involves multiple methods of data collection. We will make sure that we integrate qualitative and quantitative findings to further our understanding of the challenges associated with the implementation of the clinical programmes. Finally, we will also make sure that the research team works sufficiently close with the implementation team to be able to gather data whilst remaining independent enough to portray the problems accurately [27].

For peer review only - http://bmjopen.bmj.com/site/about/guidelines.xhtml

This study will provide us with robust new knowledge, essential for service planning, about the process of implementing these two linked programmes and the challenges to implementation success. We will also find out what patients and clinicians think of SMS and DCPC and how they can be further improved. The findings of this study will be crucial for scaling up implementation of the programmes across the UK and will enable us to construct a toolkit for this purpose. More widely, the study findings will further our understanding of the mechanisms involved in implementing new systems of care within an established clinical service.

For peer review only

For peer review only - http://bmjopen.bmj.com/site/about/guidelines.xhtml

REFERENCES

1. Torre, L.A., et al., *Global cancer statistics, 2012.* CA Cancer J Clin, 2015. **65**(2): p. 87-108.
2. Richardson, A., et al., *Knowledge, ignorance and priorities for research in key areas of cancer survivorship: findings from a scoping review.* Br J Cancer, 2011. **105 Suppl 1**: p. S82-94.
3. Walker, J. and M. Sharpe, *Depression care for people with cancer: a collaborative care intervention.* General hospital psychiatry, 2009. **31**(5): p. 436-441.
4. Strong, V., et al., *Management of depression for people with cancer (SMaRT oncology 1): a randomised trial.* The Lancet, 2008. **372**(9632): p. 40-48.
5. Sharpe, M., et al., *Integrated collaborative care for comorbid major depression in patients with cancer (SMaRT Oncology-2): a multicentre randomised controlled effectiveness trial.* The Lancet, 2014. **384**(9948): p. 1099-1108.
6. Walker, J., et al., *Integrated collaborative care for major depression comorbid with a poor prognosis cancer (SMaRT Oncology-3): a multicentre randomised controlled trial in patients with lung cancer.* The Lancet Oncology, 2014. **15**(10): p. 1168-1176.
7. Duarte, A., et al., *Cost-effectiveness of integrated collaborative care for comorbid major depression in patients with cancer.* J Psychosom Res, 2015. **79**(6): p. 465-70.
8. Walker, S., et al., *Cost-effectiveness of combining systematic identification and treatment of co-morbid major depression for people with chronic diseases: the example of cancer.* Psychol Med, 2014. **44**(7): p. 1451-60.
9. Walker, J., et al., *Prevalence, associations, and adequacy of treatment of major depression in patients with cancer: a cross-sectional analysis of routinely collected clinical data.* Lancet Psychiatry, 2014. **1**(5): p. 343-50.
10. Kroenke, K., et al., *The association of depression and pain with health-related quality of life, disability, and health care use in cancer patients.* J Pain Symptom Manage, 2010. **40**(3): p. 327-341.
11. Colleoni, M., et al., *Depression and degree of acceptance of adjuvant cytotoxic drugs.* Lancet, 2000. **356**(9238): p. 1326-1327.
12. American Psychiatric Association, *Diagnostic and Statistical Manual of Mental Mental Disorders fifth edition, DSM-5*. 2013: American Psychiatric Association.
13. First, M.B., et al., *User's guide for the structured clinical interview for DSM-IV axis I Disorders—Research version.* New York: Biometrics Research Department, New York State Psychiatric Institute, 1996.
14. Simon, G.E., D. Revicki, and M. VonKorff, *Telephone assessment of depression severity.* Journal of psychiatric research, 1993. **27**(3): p. 247-252.
15. Allen, K., A. Cull, and M. Sharpe, *Diagnosing major depression in medical outpatients: acceptability of telephone interviews.* J.Psychosom.Res., 2003. **55**(4): p. 385-387.
16. Walker, J., et al., *Screening Medical Patients for Depression: Lessons from a National Program in Cancer Clinics.* Psychosomatics, 2017.
17. Katon, W., *Collaborative depression care models: from development to dissemination.* Am J Prev Med, 2012. **42**(5): p. 550-2.
18. Hopko, D.R., et al., *Contemporary behavioral activation treatments for depression: Procedures, principles, and progress.* Clinical psychology review, 2003. **23**(5): p. 699-717.
19. Mynors-Wallis, L., *Problem-solving treatment for anxiety and depression: A practical guide*. 2005: Oxford University Press, USA.
20. Independent Cancer Taskforce, *Achieving world-class cancer outcomes. A strategy for England 2015-2020*. 2015: London.
21. Grol, R. and J. Grimshaw, *From best evidence to best practice: effective implementation of change in patients' care.* The lancet, 2003. **362**(9391): p. 1225-1230.

For peer review only

For peer review only - http://bmjopen.bmj.com/site/about/guidelines.xhtml

22. Glasgow, R.E., T.M. Vogt, and S.M. Boles, *Evaluating the public health impact of health promotion interventions: the RE-AIM framework.* American journal of public health, 1999. **89**(9): p. 1322-1327.
23. Kessler, R.S., et al., *What Does It Mean to ''Employ''the RE-AIM Model?* Evaluation & the health professions, 2012: p. 0163278712446066.
24. Pannick, S., N. Sevdalis, and T. Athanasiou, *Beyond clinical engagement: a pragmatic model for quality improvement interventions, aligning clinical and managerial priorities.* BMJ Quality & Safety, 2015.
25. Francis, J.J., et al., *What is an adequate sample size? Operationalising data saturation for theory-based interview studies.* Psychology and Health, 2010. **25**(10): p. 1229-1245.
26. Braun, V. and V. Clarke, *Using thematic analysis in psychology.* Qualitative research in psychology, 2006. **3**(2): p. 77-101.
27. Moore, G.F., et al., *Process evaluation of complex interventions: Medical Research Council guidance.* bmj, 2015. **350**: p. h1258.

For peer review only

For peer review only - http://bmjopen.bmj.com/site/about/guidelines.xhtml

AUTHOR'S CONTRIBUTIONS

Marta Wanat: study design and conduct; manuscript writing.

Jane Walker: study conception, obtained funding, study design and conduct, manuscript writing.

Katy Burke: advised on the study design, manuscript review.

Nick Sevdalis: advised on the study design, manuscript review

Alison Richardson: advised on the study design, manuscript review.

Amy Mulick: advised on the study design, manuscript review.

Chris Frost: advised on the study design, manuscript review.

Michael Sharpe: study conception, obtained funding, study design and conduct, manuscript writing.

FUNDING

This study is funded by the National Institute for Health Research (NIHR) Collaboration for Leadership in Applied Health Research and Care Oxford at Oxford Health NHS Foundation Trust. The clinical programmes are funded by Oxford University Hospitals NHS Foundation Trust in collaboration with Macmillan Cancer Support charity. Sevdalis' research is supported by the National Institute for Health Research (NIHR) Collaboration for Leadership in Applied Health Research and Care South London at King's College Hospital NHS Foundation Trust. Sevdalis is a member of King's Improvement Science, which is part of the NIHR CLAHRC South London and comprises a specialist team of improvement scientists and senior researchers based at King's College London. Its work is funded by King's Health Partners (Guy's and St Thomas' NHS Foundation Trust, King's College Hospital NHS Foundation Trust, King's College London and South London and Maudsley NHS Foundation Trust), Guy's and St Thomas' Charity, the Maudsley Charity and the Health Foundation. Richardson's research is supported by the National Institute for Health Research (NIHR) Collaboration for Leadership in Applied Health Research and Care Wessex at the University

For peer review only - http://bmjopen.bmj.com/site/about/guidelines.xhtml

Hospital Southampton NHS Foundation Trust. The views expressed are those of the authors and not necessarily those of the NHS, the NIHR, or the Department of Health.

COMPETING INTEREST

Sevdalis is the director of London Training & Safety Solutions Ltd, which consults on team training, human factors and patient safety.

For peer review only

For peer review only - http://bmjopen.bmj.com/site/about/guidelines.xhtml

BMJ Open

Linked symptom monitoring and depression treatment programmes for specialist cancer services: Protocol for a mixed-methods implementation study.

Journal:	*BMJ Open*
Manuscript ID	bmjopen-2017-016186.R2
Article Type:	Protocol
Date Submitted by the Author:	02-Jun-2017

Complete List of Authors:	Wanat, Marta; University of Oxford, Psychiatry<br>Walker, J.; University of Oxford, Psychiatry<br>Burke, Katy; University of Oxford, Psychiatry<br>Sevdalis, Nick; King's College London,<br>Richardson, Alison; University of Southampton, Faculty of Health Sciences<br>Mulick, Amy; London School of Hygiene and Tropical Medicine<br>Frost, Chris; London School of Hygiene & Tropical Medicine<br>Sharpe, Michael; University of Oxford, Psychiatry

**Primary Subject Heading**:	Health services research
Secondary Subject Heading:	Health services research
Keywords:	ONCOLOGY, Depression & mood disorders < PSYCHIATRY, implementation process, mixed methods

SCHOLARONE™ Manuscripts

For peer review only

Title: Linked symptom monitoring and depression treatment programmes for specialist cancer services: Protocol for a mixed-methods implementation study.

Authors: Marta Wanat, Jane Walker, Katy Burke, Nick Sevdalis, Alison Richardson, Amy Mulick, Chris Frost, Michael Sharpe.

Author details:

Marta Wanat*, Psychological Medicine Research, University of Oxford Department of Psychiatry, Warneford Hospital, Oxford, UK. marta.wanat@psych.ox.ac.uk, +44 01865 613106

Jane Walker, Psychological Medicine Research, University of Oxford Department of Psychiatry, Warneford Hospital, Oxford,UK. jane.walker@psych.ox.ac.uk

Katy Burke, Psychological Medicine Research, University of Oxford Department of Psychiatry, Warneford Hospital, Oxford, UK. katharine.burke@psych.ox.ac.uk

Nick Sevdalis, Centre for Implementation Science, King's College London, London, UK. nick.sevdalis@kcl.ac.uk

Alison Richardson, Faculty of Health Sciences, University of Southampton, Southampton, UK. alison.richardson@soton.ac.uk

Amy Mulick, Department of Medical Statistics, London School of Hygiene and Tropical Medicine, London, UK. Amy.Mulick@lshtm.ac.uk

Chris Frost, Department of Medical Statistics, London School of Hygiene and Tropical Medicine, London, UK. Chris.Frost@lshtm.ac.uk

Michael Sharpe, Psychological Medicine Research, University of Oxford Department of Psychiatry, Warneford Hospital, Oxford, UK. michael.sharpe@psych.ox.ac.uk

*corresponding author

Word count: 2778

For peer review only - http://bmjopen.bmj.com/site/about/guidelines.xhtml

ABSTRACT

Introduction

There is growing awareness that cancer services need to address patients' wellbeing as well as treating their cancer. We developed systematic approaches to (a) monitoring patients' symptoms including depression using a 'Symptom Monitoring Service' and (b) providing treatment for those with major depression using a programme called 'Depression Care for People with Cancer'. Used together, these two programmes were found to be highly effective and cost-effective in clinical trials. The overall aims of this project are to: (1) study the process of introducing these programmes into routine clinical care in a large cancer service, (2) identify the challenges associated with implementation and how these are overcome, (3) determine their effectiveness in a routine non-research setting, (4) describe patients' and clinicians' experience of the programmes.

Methods and analysis

This is a mixed methods longitudinal implementation study. We will study the process of implementation in three phases (April 2016-December 2018): "Pre-implementation" (setting up of the new programmes), "Early Implementation" (implementation of the programmes in a small number of clinics) and "Implementation and Maintenance" (implementation in the majority of clinics). We will use the following methods of data collection: (a) contemporaneous logs of the implementation process, (b) interviews with healthcare professionals and managers, (c) interviews with patients, (d) routinely collected clinical data.

Ethics and dissemination

The study has been reviewed by a joint committee of Oxford University Hospitals NHS Foundation Trust Research and Development Department and the University of Oxford's Clinical Trials and Research Governance Department and judged to be service evaluation, not requiring ethics committee approval. The findings of this study will guide the scaling up implementation of the

For peer review only - http://bmjopen.bmj.com/site/about/guidelines.xhtml

programmes across the UK and will enable us to construct an implementation toolkit. We will disseminate our findings in publications and at relevant national and international conferences.

Keywords Cancer care; symptoms; depression; mixed-methods; RE-AIM; implementation processes

For peer review only

For peer review only - http://bmjopen.bmj.com/site/about/guidelines.xhtml

STRENGTHS AND LIMITATIONS

- The mixed-methods design of this study will produce a comprehensive summary of the process of, and barriers and facilitators to, implementation.
- The implementation of our depression screening and treatment programmes was explicitly advocated in the 2015 NHS Cancer Taskforce report, and the findings of this study will inform the process of implementation in other centres.
- The timeline of the study may be affected by the pace of implementation.
- The study findings may not be readily generalizable to other healthcare systems.
- There may be difficulties recruiting patients who do not use SMS or DCPC, which may limit our understanding of these patients' views towards the services.

For peer review only

INTRODUCTION

World-wide, 14.1 million people are diagnosed with cancer each year [1]. Whilst there have been significant advances in anticancer treatments, leading to a longer life expectancy for many patients, there remains a pressing need to also improve patients' quality of life by better management of common cancer-related symptoms such as depression, pain and fatigue [2].

As part of a research programme, we developed two clinical programmes which aimed to improve the quality of life of patients attending cancer services. The first was a systematic approach to the assessment of symptoms, including depression, called the 'Symptom Monitoring Service' (SMS). The second was a system of care for those cancer patients identified as having depression, called 'Depression Care for People with Cancer'(DCPC) [3]. We prioritised the treatment of depression as its management is especially challenging for cancer services. Our research found that, used together, SMS and DCPC are highly effective and cost-effective in improving the lives of those cancer patients who suffer from comorbid depression [4-8].

Symptom Monitoring Service (SMS)

The SMS provides a systematic way of monitoring patients' symptoms, including depression, every time they attend the clinic. Approximately 10% of cancer outpatients have comorbid major depression [9]. Depression substantially reduces patients' quality of life and ability to cope with anticancer treatments, but often goes undetected and untreated [9-11]. Patients using the SMS are assisted to complete brief questionnaires about relevant symptoms using touchscreen computers whilst waiting for their appointment. These questionnaires include symptoms relevant to all clinics (e.g. depressive symptoms and pain) as well as symptoms relevant to specific clinics (e.g. coughing up blood in the lung cancer clinic). The results of these questionnaires are available to the patient's clinician (e.g. oncologist or cancer nurse specialist) before the appointment. The clinician can use this information to: (a) identify patients with severe symptoms; (b) detect changes in patients'

For peer review only - http://bmjopen.bmj.com/site/about/guidelines.xhtml

symptoms over time; (c) determine which symptoms require further assessment; (d) ensure that patients receive appropriate treatment for their symptoms. To identify patients with major depression (the diagnostic term for depression which requires treatment), those with a high score on the relevant symptom questionnaire are offered a telephone-delivered diagnostic interview [12]. These semi-structured clinical interviews are based on the major depression section of the Structured Clinical Interview for the Diagnostic and Statistical Manual of Mental Disorders) [13]. Telephone interviews are used because these are more feasible than attempting to achieve interviews in the clinic, are convenient and acceptable to patients, and have been found to have good agreement with face-to-face interviews [14, 15]. The SMS is delivered by a team of symptom monitoring assistants, trained and supervised by consultation-liaison psychiatrists; further details are given in our previous publication[16]. Patients identified as having a diagnosis of major depression are offered participation in the linked 'Depression Care for People with Cancer' treatment programme.

Depression Care for People with Cancer (DCPC)

DCPC is a manualised, systematic, multi-component, team-delivered treatment programme for patients with cancer and major depression that is based on the collaborative care model [3, 17]. The treatment team comprises care managers (specially trained cancer nurses) and consultation-liaison psychiatrists who work closely with the patient's GP and oncologist. The care managers establish a therapeutic relationship with the patient, provide education about depression, deliver brief evidence-based psychological treatments (behavioural activation and problem solving therapy) and systematically monitor the patient's progress [18, 19]. The psychiatrists supervise treatment with the aim of achieving and then maintaining specified treatment targets, advise GPs and oncologists on the prescription of antidepressant medication and also provide direct consultations to those patients who are not improving.

For peer review only - http://bmjopen.bmj.com/site/about/guidelines.xhtml

The process of implementing a new service

Implementation of these systems has been explicitly advocated in the 2015 NHS Cancer Taskforce report [20]. However, it is well known that the translation from delivering services as part of research programmes to delivering them in routine clinical practice can be difficult and may lead to innovative services failing to realise their promise [21].

Aim

Our aim is to study the implementation of SMS and DCPC into the routine clinical care provided by a large cancer service in Oxford, UK, with a view to learning how best to facilitate the introduction of these programmes into other cancer services. We will: (1) describe the process of implementation, (2) identify the challenges in implementation and how these are overcome, (3) determine the programmes' effectiveness in clinical practice and (4) describe patients' and clinicians' experience of them. Detailed aims are described in Table 1.

For peer review only

For peer review only - http://bmjopen.bmj.com/site/about/guidelines.xhtml

METHODS AND ANALYSIS

Study design

We will use a mixed-methods design to longitudinally study the process of introducing SMS and DCPC into the Oxford Cancer Centre. The Oxford Cancer Centre is a large cancer service, which is part of Oxford University Hospitals NHS Foundation Trust. It receives approximately 19,000 new referrals per year, has 58 consultant oncologists and comprises inpatient wards, operating theatres, a day hospital and outpatient departments.

The study will focus on three phases: "Pre-implementation" (setting up of the new programmes), "Early Implementation" (implementation in a small number of clinics) and "Implementation and Maintenance" (implementation in the majority of clinics). Our specific aims are described in detail in Table 1. In the pre-implementation phase we will study the process of setting up the new programmes, the challenges encountered during set up and the ways these are addressed. In the early implementation and implementation and maintenance phases, we will describe the challenges encountered and how they are addressed, the effectiveness of the programmes in clinical practice and the experiences of both the patients who have access to SMS and DCPC and of the healthcare professionals involved in them.

For peer review only

Table 1 Study aims related to each phase of implementation

Phase of implementation	Study aim	Relevant Re-aim components	Data collection methods
Pre-implementation	Describe the timeline and process of setting up the new programmes in the cancer service	N/A	Contemporaneous log
	Describe the challenges of setting up the programmes in the cancer service & the ways these are addressed including the experiences of the health professionals and managers involved in set up	N/A	Contemporaneous log, interviews with healthcare professionals & managers
Early implementation	Describe the timeline and process of introducing SMS & DCPC in a small number of clinics	Implementation, Adoption	Contemporaneous log
	Describe the challenges associated with day-to-day running & how these are addressed	Implementation, Adoption, Effectiveness	Contemporaneous log, interviews with healthcare professionals
	Describe the experiences of healthcare professionals involved in SMS & DCPC	Implementation, Adoption, Effectiveness, Reach	Interviews with healthcare professionals
	Describe patients' experience of SMS & DCPC & how these affect their use of them	Effectiveness, Reach	Interviews with patients
	Measure the clinical effectiveness of DCPC by comparing patient outcomes with those achieved in clinical trials	Effectiveness	Routine clinical data
	Measure the proportion & characteristics of the target population who have access to & who use SMS & DCPC	Reach	Routine clinical data
Implementation and maintenance	Describe the timeline and process of the introduction of SMS & DCPC in the majority of clinics	Adoption, Maintenance	Contemporaneous log
	Describe the challenges associated with implementation and long-term maintenance & how these are addressed	Implementation, Maintenance	Contemporaneous log, interviews with healthcare professionals
	Describe the challenges of delivering SMS & DCPC as intended & adaptations made to the programmes	Implementation, maintenance	Contemporaneous log, interviews with healthcare professionals
	Describe the experiences of healthcare professionals involved in SMS & DCPC	Adoption, Effectiveness	Interviews with healthcare professionals
	Describe patients' experience of SMS & DCPC & how these affect their use of them	Effectiveness, Reach	Interviews with patients
	Measure the effectiveness of DCPC over time by comparing patient outcomes with those achieved in clinical trials	Effectiveness, Maintenance	Routine clinical data
	Determine the extent to which SMS & DCPC are delivered as intended	Implementation	Routine clinical data
	Measure the proportion & characteristics of the target population who have access to & who use SMS & DCPC over time	Reach	Routine clinical data
	Measure the cost of delivering SMS & DCPC	Implementation	Routine clinical data

For peer review only

For peer review only - http://bmjopen.bmj.com/site/about/guidelines.xhtml

We will use the following methods of data collection: (a) contemporaneous logs of the implementation process, (b) interviews with healthcare professionals and managers, (c) interviews with patients, (d) routine clinical data. Data collection in the Early Implementation and Implementation and Maintenance phases will be guided by the RE-AIM (Reach, Effectiveness, Adoption, Implementation, Maintenance) framework [22, 23].

Pre-implementation phase

Contemporaneous log

A contemporaneous events log will be used by the study team to record any activities and events related to setting up the new programmes in the cancer service and will include regular correspondence from staff involved in setting up the service [24]. Sample activities and events to be included are recruitment and training events, and meetings with clinical managers and IT experts. Each entry to the log will include information on the date of the action or event, the people involved and its effects on the delivery of the programmes.

Interviews with healthcare professionals and managers

We will recruit and interview 10-15 healthcare professionals and managers who are involved in setting up SMS and DCPC in the cancer service. Based on similar studies, we anticipate this sample size will be sufficient to reach data saturation [25]. Interviews will take approximately 30 minutes, will be audio-recorded and transcribed verbatim and will be conducted at the participants' preferred location or by telephone. Interviews will be semi-structured following a topic guide which will focus on participants' views of the process of setting up the programmes. They will conducted by a trained member of the study team with previous experience of interviewing healthcare professionals.

For peer review only - http://bmjopen.bmj.com/site/about/guidelines.xhtml

Analysis

A timeline of events that occur during set up will be drawn using the contemporaneous log. Data from the log will then be analysed using content analysis, which will involve data familiarisation and coding. Codes will be developed focusing on the process of setting up the programmes, the associated challenges and the ways that these are addressed. The codes developed from the contemporaneous log will be applied to the transcripts of interviews with healthcare professionals and managers and will be further refined. These codes will be then sorted into subcategories and categories.

Early Implementation and Implementation and Maintenance phases

Contemporaneous log

We will record and use data in a contemporaneous events log in the same away as in the pre-implementation phase. This will allow us to describe the time taken for each step of implementation.

Interviews with healthcare professionals and managers

We will recruit 20-30 healthcare professionals and managers during the Early Implementation and Implementation and Maintenance phases. These will include psychiatrists and care managers involved in the delivery of the SMS and DCPC, and oncologists, cancer nurse specialists and GPs who make up the wider collaborative care team. Based on similar studies, we anticipate this sample size will be sufficient to reach data saturation [25]. Semi-structured Interviews will take approximately 30 minutes, will be audio-recorded and transcribed verbatim and will be conducted at the participant's preferred location or by telephone. Interviews will be conducted by a trained member of the study team with previous experience of interviewing healthcare professionals. The interview topic guide will focus on the participants' experience of the new programmes.

For peer review only - http://bmjopen.bmj.com/site/about/guidelines.xhtml

For peer review only

Interviews with patients

We will recruit and interview 40-60 patients during the Early Implementation and Implementation and Maintenance phases. These will include patients who do not use the first stage of the SMS service (n=10-15), who use the SMS service (n=10-15), who do not use the DCPC treatment programme (n=10-15), and who use the DCPC treatment programme (n=10-15). We will exclude patients who are unable to provide informed consent to participate or do not understand and speak English sufficiently to participate in a qualitative interview. Interviews will take between 30-60 minutes, will be audio-recorded and transcribed verbatim and will be conducted at the participant's preferred location or by telephone. Interviews will be conducted by a trained member of the study team with previous experience of interviewing patients with cancer. The interview topic guide will focus on patients' understanding and views of the SMS and DCPC.

Routine clinical data

We will obtain the following data from the cancer centre: the number and characteristics of clinics with and without the new programmes over time, the proportion of patients who do and do not receive each part of the programmes and their characteristics, the staff and other resources required and costs of delivering SMS and DCPC, adherence to the SMS and DCPC manuals including diagnostic accuracy, outcomes of patients who receive the programmes and description of any relevant adverse clinical events.

Analysis

Data recorded in the contemporaneous log related to the introduction of SMS and DCPC in clinics will be used to draw a timeline across the Early Implementation and Implementation and Maintenance phases. Then, data from the contemporaneous log will be analysed using content analysis, which will involve data familiarisation and coding. Codes will be developed focusing on: the process of implementation, challenges associated with day-to-day running of SMS and DCPC,

For peer review only - http://bmjopen.bmj.com/site/about/guidelines.xhtml

challenges associated with implementation and long-term maintenance, challenges associated with delivering SMS and DCPC as intended, and the ways that these challenges are addressed. Codes developed from the contemporaneous log will be applied to selected relevant excerpts from the transcripts of qualitative interviews with healthcare professionals. These codes will be further refined and then sorted into subcategories and categories.

Data generated from the qualitative interviews with patients and with healthcare professionals involved in SMS and DCPC will be analysed using thematic analysis to describe their experiences [26]. The stages of the analysis will include data familiarisation, coding and development of themes. Data collection and analysis will take place simultaneously to allow the exploration of themes from the initial interviews in subsequent ones. In order to minimise subjective biases, the coding will be discussed among members of the study team.

We will analyse the routine clinical data using descriptive statistics in order to summarise: the effectiveness of DCPC over time (including benchmarking against the patient outcomes in our clinical trials), the proportion and characteristics of the target population who have access to and who use SMS and DCPC over time, the fidelity of the delivery of SMS and DCPC, adherence of patients with each component of the DCPC and the cost of the new services. We will use data from qualitative interviews to understand these results, for example how challenges in the adherence to the protocol may influence the effectiveness of the service.

Ethics and dissemination

The study has been reviewed by a joint committee of Oxford University Hospitals NHS Foundation Trust Research and Development Department and the University of Oxford's Clinical Trials and Research Governance Department and been judged to be service evaluation, not requiring ethics

For peer review only - http://bmjopen.bmj.com/site/about/guidelines.xhtml

For peer review only

committee approval. The study has been registered with the Oxford University Hospitals NHS Foundation Trust service evaluation and clinical audit team. The results of the study will be disseminated via presentations at local, national and international conferences, peer-reviewed journals and workshops with stakeholders. The implementation of SMS and DCPC in UK cancer centres is explicitly advocated in the 2015 NHS Cancer Taskforce report [10]. The findings from the study will be crucial for scaling up implementation and will form part of a toolkit for doing this.

For peer review only

For peer review only - http://bmjopen.bmj.com/site/about/guidelines.xhtml

DISCUSSION

Our overall aim is to study the process of setting up and introducing two new linked clinical programmes (SMS and DCPC) in a large UK cancer service, to describe the challenges associated with their implementation and how these are addressed, to measure their effectiveness in clinical practice and to describe patients' and clinicians' experience of using them. Whilst these programmes have already been successfully implemented in the context of a large research programme and have been found to be effective and cost-effective in clinical trials, this is first time that they have been implemented in a non-research setting.

The Early Implementation and Implementation and Maintenance phases of our study will use the RE-AIM framework to guide data collection, analysis and reporting of findings. The authors of this framework have recently highlighted the importance of collecting data in relation to all components of RE-AIM framework and especially qualitative data about patients not using the service [23]. Our study follows both of these recommendations.

We anticipate a number of challenges in conducting this research. Firstly, the timeline of the study will be affected by the pace of implementation. We have divided the study into three phases to minimise delays to data collection. Secondly, there may be difficulties in recruiting busy healthcare professionals and managers for interviews. We have mitigated this risk by working closely with healthcare professionals and managers to identify research questions which are of interest to them. Thirdly, this study involves multiple methods of data collection. We will make sure that we integrate qualitative and quantitative findings to further our understanding of the challenges associated with the implementation of the clinical programmes. Finally, we will also make sure that the research team works sufficiently close with the implementation team to be able to gather data whilst remaining independent enough to portray the problems accurately [27].

For peer review only - http://bmjopen.bmj.com/site/about/guidelines.xhtml

This study will provide us with robust new knowledge, essential for service planning, about the process of implementing these two linked programmes and the challenges to implementation success. We will also find out what patients and clinicians think of SMS and DCPC and how they can be further improved. The findings of this study will be crucial for scaling up implementation of the programmes across the UK and will enable us to construct a toolkit for this purpose. More widely, the study findings will further our understanding of the mechanisms involved in implementing new systems of care within an established clinical service.

For peer review only

For peer review only - http://bmjopen.bmj.com/site/about/guidelines.xhtml

REFERENCES

1. Torre, L.A., et al., *Global cancer statistics, 2012.* CA Cancer J Clin, 2015. **65**(2): p. 87-108.
2. Richardson, A., et al., *Knowledge, ignorance and priorities for research in key areas of cancer survivorship: findings from a scoping review.* Br J Cancer, 2011. **105 Suppl 1**: p. S82-94.
3. Walker, J. and M. Sharpe, *Depression care for people with cancer: a collaborative care intervention.* General hospital psychiatry, 2009. **31**(5): p. 436-441.
4. Strong, V., et al., *Management of depression for people with cancer (SMaRT oncology 1): a randomised trial.* The Lancet, 2008. **372**(9632): p. 40-48.
5. Sharpe, M., et al., *Integrated collaborative care for comorbid major depression in patients with cancer (SMaRT Oncology-2): a multicentre randomised controlled effectiveness trial.* The Lancet, 2014. **384**(9948): p. 1099-1108.
6. Walker, J., et al., *Integrated collaborative care for major depression comorbid with a poor prognosis cancer (SMaRT Oncology-3): a multicentre randomised controlled trial in patients with lung cancer.* The Lancet Oncology, 2014. **15**(10): p. 1168-1176.
7. Duarte, A., et al., *Cost-effectiveness of integrated collaborative care for comorbid major depression in patients with cancer.* J Psychosom Res, 2015. **79**(6): p. 465-70.
8. Walker, S., et al., *Cost-effectiveness of combining systematic identification and treatment of co-morbid major depression for people with chronic diseases: the example of cancer.* Psychol Med, 2014. **44**(7): p. 1451-60.
9. Walker, J., et al., *Prevalence, associations, and adequacy of treatment of major depression in patients with cancer: a cross-sectional analysis of routinely collected clinical data.* Lancet Psychiatry, 2014. **1**(5): p. 343-50.
10. Kroenke, K., et al., *The association of depression and pain with health-related quality of life, disability, and health care use in cancer patients.* J Pain Symptom Manage, 2010. **40**(3): p. 327-341.
11. Colleoni, M., et al., *Depression and degree of acceptance of adjuvant cytotoxic drugs.* Lancet, 2000. **356**(9238): p. 1326-1327.
12. American Psychiatric Association, *Diagnostic and Statistical Manual of Mental Mental Disorders fifth edition, DSM-5*. 2013: American Psychiatric Association.
13. First, M.B., et al., *User's guide for the structured clinical interview for DSM-IV axis I Disorders—Research version.* New York: Biometrics Research Department, New York State Psychiatric Institute, 1996.
14. Simon, G.E., D. Revicki, and M. VonKorff, *Telephone assessment of depression severity.* Journal of psychiatric research, 1993. **27**(3): p. 247-252.
15. Allen, K., A. Cull, and M. Sharpe, *Diagnosing major depression in medical outpatients: acceptability of telephone interviews.* J.Psychosom.Res., 2003. **55**(4): p. 385-387.
16. Walker, J., et al., *Screening Medical Patients for Depression: Lessons from a National Program in Cancer Clinics.* Psychosomatics, 2017.
17. Katon, W., *Collaborative depression care models: from development to dissemination.* Am J Prev Med, 2012. **42**(5): p. 550-2.
18. Hopko, D.R., et al., *Contemporary behavioral activation treatments for depression: Procedures, principles, and progress.* Clinical psychology review, 2003. **23**(5): p. 699-717.
19. Mynors-Wallis, L., *Problem-solving treatment for anxiety and depression: A practical guide*. 2005: Oxford University Press, USA.
20. Independent Cancer Taskforce, *Achieving world-class cancer outcomes. A strategy for England 2015-2020*. 2015: London.
21. Grol, R. and J. Grimshaw, *From best evidence to best practice: effective implementation of change in patients' care.* The lancet, 2003. **362**(9391): p. 1225-1230.

For peer review only - http://bmjopen.bmj.com/site/about/guidelines.xhtml

For peer review only

22. Glasgow, R.E., T.M. Vogt, and S.M. Boles, *Evaluating the public health impact of health promotion interventions: the RE-AIM framework.* American journal of public health, 1999. **89**(9): p. 1322-1327.
23. Kessler, R.S., et al., *What Does It Mean to ''Employ''the RE-AIM Model?* Evaluation & the health professions, 2012: p. 0163278712446066.
24. Pannick, S., N. Sevdalis, and T. Athanasiou, *Beyond clinical engagement: a pragmatic model for quality improvement interventions, aligning clinical and managerial priorities.* BMJ Quality & Safety, 2015.
25. Francis, J.J., et al., *What is an adequate sample size? Operationalising data saturation for theory-based interview studies.* Psychology and Health, 2010. **25**(10): p. 1229-1245.
26. Braun, V. and V. Clarke, *Using thematic analysis in psychology.* Qualitative research in psychology, 2006. **3**(2): p. 77-101.
27. Moore, G.F., et al., *Process evaluation of complex interventions: Medical Research Council guidance.* bmj, 2015. **350**: p. h1258.

For peer review only

AUTHOR'S CONTRIBUTIONS

Marta Wanat: study design and conduct; manuscript writing.

Jane Walker: study conception, obtained funding, study design and conduct, manuscript writing.

Katy Burke: advised on the study design, manuscript review.

Nick Sevdalis: advised on the study design, manuscript review

Alison Richardson: advised on the study design, manuscript review.

Amy Mulick: advised on the study design, manuscript review.

Chris Frost: advised on the study design, manuscript review.

Michael Sharpe: study conception, obtained funding, study design and conduct, manuscript writing.

FUNDING

This study is funded by the National Institute for Health Research (NIHR) Collaboration for Leadership in Applied Health Research and Care Oxford at Oxford Health NHS Foundation Trust. The clinical programmes are funded by Oxford University Hospitals NHS Foundation Trust in collaboration with Macmillan Cancer Support charity. Sevdalis' research is supported by the National Institute for Health Research (NIHR) Collaboration for Leadership in Applied Health Research and Care South London at King's College Hospital NHS Foundation Trust. Sevdalis is a member of King's Improvement Science, which is part of the NIHR CLAHRC South London and comprises a specialist team of improvement scientists and senior researchers based at King's College London. Its work is funded by King's Health Partners (Guy's and St Thomas' NHS Foundation Trust, King's College Hospital NHS Foundation Trust, King's College London and South London and Maudsley NHS Foundation Trust), Guy's and St Thomas' Charity, the Maudsley Charity and the Health Foundation. Richardson's research is supported by the National Institute for Health Research (NIHR) Collaboration for Leadership in Applied Health Research and Care Wessex at the University

For peer review only - http://bmjopen.bmj.com/site/about/guidelines.xhtml

Hospital Southampton NHS Foundation Trust. The views expressed are those of the authors and not necessarily those of the NHS, the NIHR, or the Department of Health.

COMPETING INTEREST

Sevdalis is the director of London Training & Safety Solutions Ltd, which consults on team training, human factors and patient safety.

For peer review only

For peer review only - http://bmjopen.bmj.com/site/about/guidelines.xhtml
